# Is It Time to Cross the Pillars of Evidence in Favor of Segmentectomies in Early-Stage Non-Small Cell Lung Cancer?

**DOI:** 10.3390/cancers15071993

**Published:** 2023-03-27

**Authors:** Luca Bertolaccini, Lorenzo Spaggiari

**Affiliations:** 1 Department of Thoracic Surgery, IEO, European Institute of Oncology IRCCS, 20141 Milan, Italy; lorenzo.spaggiari@ieo.it; 2 Department of Oncology and Hemato-Oncology, University of Milan, 20122 Milan, Italy

**Keywords:** lung cancer, lobectomy, segmentectomy, randomized controlled trial

## 1. Introduction

In the debate on lobectomy versus segmentectomy for the treatment of early-stage non-small cell lung cancer (NSCLC), currently, we have reached two pillars of knowledge, like Jachim and Boaz, which have encompassed the actual boundary of the literature published up until now. Twenty-six years ago, the North American Lung Cancer Study Group revealed results indicating superior overall survival following lobectomy in comparison to typical or atypical segmentectomy for early-stage NSCLC [1]. Last year, the publication in The Lancet of the Japanese randomized control trial, JCOG0802/WJOG4607L, was the first phase III trial to show the benefits of segmentectomy versus lobectomy in terms of overall survival [2]. Based on this conclusion, anatomical (typical) segmentectomy should become the standard surgical procedure for stage IA NSCLC as opposed to lobectomy.

In addition, based on the randomized controlled trial CALGB 140503, which was published this year in *The New England Journal of Medicine*, in clinical T1aN0 non-small cell lung cancer, sublobar resections (wedge resection or anatomical segmentectomy) are successful therapeutic approaches [3].

Unlike lobectomies, for which few technical variations have been described, segmentectomies have a plethora of different interventions. Segmentectomies can include different functional and technical oncological aspects that complicate the results’ homogenization. Therefore, the evidence reported suggests a cross-over between the two pillars and the adoption of segmentectomy as the standard of care. On the other hand, after reading the aforementioned innovative papers, the superiority of segmentectomy still remains, in our opinion, controversial, and there are five points that deserve further discussion: the different techniques of performing segmentectomies with particular attention paid to complex resection, functional aspects, local recurrence, and long-term cancer-specific survival.

## 2. Segmentectomy Does Not Seem to Preserve Lung Function

The first question to be answered is what factors indicate the recommendation for segmentectomy in a patient with early-stage lung cancer, and the answer should be to preserve better pulmonary function than is the case following lobectomy. From a theoretical point of view, segmentectomies should be anatomically and functionally superior to lobectomies, according to the minimal evidence published in the literature. Due to the inability of the adult lung to regenerate new alveolar septal tissues, postoperative pulmonary function is mainly determined by the volume of the lung removed. Following lobectomy, the non-operated lobe(s) exhibits anatomical excursion. The modification in forced expiratory volume (FEV1) in the first second, measuring airway resistance, is predominantly attributable to ventilation mechanisms (e.g., existing airway obstruction, compensatory expansion of the residual lung, and chest wall activity). A lobectomy will ultimately result in the displacement of the remaining lobe(s). The decrease in FEV1 is more significant in lobectomies, suggesting that lobectomies are more likely to increase airway resistance. The amount of resected lung tissue mainly determines changes in forced vital capacity (FVC), and the remaining portion of the lung swells and compensates for the removed lobe. Diffuse lung carbon oxide (DLCO) represents the accessible capillary surface area for gas diffusion; segmentectomies have a lower degree of DLCO decrease, indicating better preservation of oxygenation and protection of lung function in comparison to lobectomies [4].

On the other hand, the JCOG0802/WJOG4607L trial did not find the expected evidence of superiority in postoperative respiratory function in segmentectomies. Consequently, less lung parenchyma excision would not necessarily result in improved function preservation, which could be caused by less acceptable re-expansion of the residual lobe following segmentectomy. In addition, the remaining lobe(s) in the ipsilateral or contralateral lung following lobectomy expands and compensates better than is the case following segmentectomy [5]. Therefore, it is thus far too early to define segmentectomy as the standard of care before we know in what way segmentectomies would benefit which subsets of patients [6].

## 3. The Surgical Difficulties of Different Forms of Segmentectomy Are Dissimilar

The JCOG0802/WJOG4607L trial excluded basal segmentectomy, which involves the removal of all segments except the apical segment S6 in the lower lobe because less lung parenchymal tissue is preserved. On the other hand, the same trial accepted apical trisegmentectomy [2]. The JCOG0802/WJOG4607L trial also did not stratify the outcomes regarding the different types of segmentectomies. It is well known to thoracic surgeons that the different types of segmentectomies described are not the same in terms of operational difficulty [7]. The anatomical challenges of segmentectomies, especially in the case of non-palpable tumors, may hinder the achievement of negative resection margins and radical hilar lymph node dissection. The literature classifies segmentectomies in terms of *simple* or *complex* procedures based on the number and shape of intersegmental planes (Table 1) [8].

Another factor to consider is the technical difficulty of identifying small nodules and ensuring proper margin distances in segmentectomies (amplified in pure ground-glass opacity) [10]. In the JCOG0802/WJOG4607L trial, even if more locoregional relapses occurred in segmentectomies, the total relapse pattern (distant relapse and both distant and locoregional relapse) was comparable between the approaches [2]. In segmentectomies, ipsilateral or contralateral mediastinal lymph node recurrence occurred more frequently than in the lobectomies during the first relapse. Therefore, a complete investigation of radiological and pathological findings and surgical techniques is essential to comprehend how different types of segmentectomies might be improved [2].

## 4. Lymph Node Mediastinal Dissection Still Remains a Key Point

In the JCOG0802/WJOG4607L trial, the mediastinal lymph nodes should have been dissected systematically, but selective dissection was also permitted and accepted [2]. Earlier evidence in the literature showed that systematic lymph node dissection yielded more metastatic lymph nodes and better oncologic outcomes than lobe-specific lymph node dissection [11]. Immune checkpoint inhibitors and tyrosine kinase inhibitors therapy are the topics of ongoing investigations in both the neoadjuvant and adjuvant settings for resectable NSCLC. A multimodal strategy has become the standard of care, particularly for hypermetabolic malignancies with a higher propensity for nodal metastasis. Therefore, systematic nodal dissection ensures appropriate staging, critical for selecting patients for adjuvant treatment. In light of emerging novel systemic treatments, inaccurate nodal sampling may inappropriately deprive patients of these effective treatments, impacting their survival [12].

## 5. Wedge Resections and Anatomical Segmentectomies Are Not Two Sides of the Same Coin

The randomized controlled trial CALGB140503 was based on 357 lobectomies and 340 sublobar resections (59.1% wedge resection versus 37.9% anatomical segmentectomy).

Wedge resections were noted in the sublobar resection group as being the most frequently practiced approach in Europe and North America [3]. This inclusion was carried out by researchers referring to a real-world setting, but it was based on databases prior to 2013 [13,14].

The Society of Thoracic Surgeons General Thoracic Surgery Database, one of the world’s most comprehensive surgical databases, collected data on the proportion of the type of resections performed from 2013 to 2020. Notably, wedge resections were not the most frequently performed form of surgery over this period, and they also decreased from 36% to 30%, with an increase from 51% to 55% being noted for lobectomies [15].

## 6. The Design of a Randomized Controlled Trial

Clinicians regularly read and evaluate randomized controlled trials to inform their practice, but how can they be sure that such randomized controlled trials are accurate and trustworthy? Not all randomized controlled trials are the same; therefore, caution must be taken when deciding if randomized controlled trial results warrant a change in future patient management. The most effective method for assessing the validity of a randomized controlled trial is to consider the potential risks of bias associated with that study. A study is biased when a component of its design or execution has systemic effects on its results that differ from the truth. When such a bias exists, a study may result in an overestimation or underestimation of the truth, jeopardizing the validity of its findings or conclusions, even if all other aspects of the investigation were adequate. Inaccurate results may be provided to doctors and patients, notwithstanding the thoroughness of the investigation, by an otherwise solid study that contains some bias. In light of this, it is essential to comprehend the forms of bias that may occur within randomized controlled trials, how to detect these potential biases, and how to interpret the results in light of these potential biases [16].

Therefore, the nature of the chosen intervention can significantly impact the results produced. The phase at which an intervention is examined can be crucial. Too early bias and too late bias can influence the observed effects. This is especially true for surgical trials, in which there may be a learning curve bias for novel operators or improvements (or regressions) in the approaches or situations in which they are utilized. Complexity bias can arise when a trial examines interventions with several components or when the outcomes depend on multiple factors beyond the investigator’s control.

## 7. Conclusions

The randomized study JCOG0802/WJOG4607L suggests that segmentectomy should replace lobectomy as the standard surgical treatment for patients with small (<2 cm) peripheral clinical stage IA NSCLC. Nevertheless, this evidence cannot lead us safely go through the pillars of segmentectomy as the gold standard [2]. We suggest two subgroups of Kaplan–Meyer estimates to researchers: cancer-specific survival and overall survival of the different subtypes of segmentectomies.

In addition, anatomical segmentectomies, removing the segmentary lymphatic pathways, are accepted as radical oncological treatments [2]. Therefore, the disease-free survival and overall survival of patients undergoing wedge resections compared to lobectomies should be added to the results of CALGB140503 [3] since this evidence could change the clinical practice management of early-stage lung cancer.

Future research should, in our opinion, apply randomized controlled trials of anatomical segmentectomies (stratified for complexity) and lobectomies to investigate the effects on outcome differences, and the appropriate cost-effectiveness analyses should be conducted.

## Figures and Tables

**Table 1:** Classification of simple and complex segmentectomies [9].

	Simple Segmentectomies	Complex Segmentectomies	
		Moderately Difficult	Difficult
**Left lung**	S1 + S2 + S3 S4 + S5 S6	S1 + S2 + S3 S1 + S2 S3 S8 S8 + S9 + S10	S8 + S9 S6 + S8
**Right lung**	S1 S6	S2 S3 S4 S8 S7 + S8 + S9 + S10	S1a + S2 S2b + S3a S2 + S3a S1b + S3 S10 S9 + S10 S8 + S9

## References

[1] Ginsberg R.J., Rubinstein L.V. (1995). Randomized trial of lobectomy versus limited resection for T1 N0 non-small cell lung cancer. Lung Cancer Study Group. Ann. Thorac. Surg..

[2] Saji H., Okada M., Tsuboi M., Nakajima R., Suzuki K., Aokage K., Aoki T., Okami J., Yoshino I., Ito H. (2022). Segmentectomy versus lobectomy in small-sized peripheral non-small-cell lung cancer (JCOG0802/WJOG4607L): A multicentre, open-label, phase 3, randomised, controlled, non-inferiority trial. Lancet.

[3] Altorki N., Wang X., Kozono D., Watt C., Landrenau R., Wigle D., Port J., Jones D.R., Conti M., Ashrafi A.S. (2023). Lobar or Sublobar Resection for Peripheral Stage IA Non-Small-Cell Lung Cancer. N. Engl. J. Med..

[4] Xu Y., Qin Y., Ma D., Liu H. (2022). The impact of segmentectomy versus lobectomy on pulmonary function in patients with non-small-cell lung cancer: A meta-analysis. J. Cardiothorac. Surg..

[5] Li H., Shen C., Chen Y., Wang Y., Zhong C., Fang W. (2022). What Do We Talk About Now When We Talk About Segmentectomy for GGO?. Front. Surg.

[6] Fang W. (2020). Commentary: Is segmentectomy ready to be accepted as the standard of care?. J. Thorac. Cardiovasc. Surg..

[7] Wu H., Zhang Y., Li H. (2020). Robotic complex segmentectomies. Video-Assist. Thorac. Surg..

[8] Bertolaccini L., Spaggiari L. (2022). Commentary: The sublobar resections and the difference between a conjecture and a theorem. J. Thorac. Cardiovasc. Surg..

[9] Oizumi H., Kanauchi N., Kato H., Endoh M., Suzuki J., Fukaya K., Sadahiro M. (2011). Anatomic thoracoscopic pulmonary segmentectomy under 3-dimensional multidetector computed tomography simulation: A report of 52 consecutive cases. J. Thorac. Cardiovasc. Surg..

[10] Mazzella A., Spaggiari L. (2022). Invited commentary: Indigo carmine and lipiodol mixture marking in lung segmentectomy. Eur. J. Cardiothorac. Surg..

[11] Handa Y., Tsutani Y., Mimae T., Miyata Y., Ito H., Shimada Y., Nakayama H., Ikeda N., Okada M. (2021). Systematic Versus Lobe-Specific Mediastinal Lymphadenectomy for Hypermetabolic Lung Cancer. Ann. Surg. Oncol..

[12] Gooseman M.R., Brunelli A. (2021). Intraoperative Lymph Node Management During Non-small Cell Lung Cancer Surgery. Ann. Surg. Oncol..

[13] Seder C.W., Salati M., Kozower B.D., Wright C.D., Falcoz P.E., Brunelli A., Fernandez F.G. (2016). Variation in Pulmonary Resection Practices between the Society of Thoracic Surgeons and the European Society of Thoracic Surgeons General Thoracic Surgery Databases. Ann. Thorac. Surg..

[14] Boffa D.J., Allen M.S., Grab J.D., Gaissert H.A., Harpole D.H., Wright C.D. (2008). Data from The Society of Thoracic Surgeons General Thoracic Surgery database: The surgical management of primary lung tumors. J. Thorac. Cardiovasc. Surg..

[15] Servais E.L., Blasberg J.D., Brown L.M., Towe C.W., Seder C.W., Onaitis M.W., Block M.I., David E.A. (2023). The Society of Thoracic Surgeons General Thoracic Surgery Database: 2022 Update on Outcomes and Research. Ann. Thorac. Surg..

[16] Phillips M.R., Kaiser P., Thabane L., Bhandari M., Chaudhary V., Retina Evidence Trials InterNational Alliance Study G. (2022). Risk of bias: Why measure it, and how?. Eye.

